# A Review of Extraction and Analysis of Bioactives in Oat and Barley and Scope for Use of Novel Food Processing Technologies

**DOI:** 10.3390/molecules200610884

**Published:** 2015-06-12

**Authors:** Nirupama Gangopadhyay, Mohammad B. Hossain, Dilip K. Rai, Nigel P. Brunton

**Affiliations:** 1 Department of Food BioSciences, Teagasc Food Research Centre Ashtown, Dublin 15, Ireland; E-Mails: nirupama.gangopadhyay@teagasc.ie (N.G.); mohammad.hossain@teagasc.ie (M.B.H.); 2 Food Science and Nutrition, School of Agriculture and Food Science, University College Dublin, Dublin 4, Ireland; E-Mail: nigel.brunton@ucd.ie

**Keywords:** oat, barley, bioactives, extraction, conventional, novel food processing

## Abstract

Oat and barely are cereal crops mainly used as animal feed and for the purposes of malting and brewing, respectively. Some studies have indicated that consumption of oat and barley rich foods may reduce the risk of some chronic diseases such as coronary heart disease, type II diabetes and cancer. Whilst there is no absolute consensus, some of these benefits may be linked to presence of compounds such as phenolics, vitamin E and β-glucan in these cereals. A number of benefits have also been linked to the lipid component (sterols, fatty acids) and the proteins and bioactive peptides in oats and barley. Since the available evidence is pointing toward the possible health benefits of oat and barley components, a number of authors have examined techniques for recovering them from their native sources. In the present review, we summarise and examine the range of conventional techniques that have been used for the purpose of extraction and detection of these bioactives. In addition, the recent advances in use of novel food processing technologies as a substitute to conventional processes for extraction of bioactives from oats and barley, has been discussed.

## 1. Introduction

Cereals have been associated with food and drinks throughout the history of mankind, and they serve as a major source of energy for millions of people worldwide, to this day. In addition, consumption of whole grain cereals has long been considered to be beneficial to human health. For example, in the early 1970s, Trowell [1] in a review presented data from various epidemiological and dietary intervention studies that supported the hypothesis that a diet consisting of whole grain cereals may decrease the incidence of ischemic heart disease and hyperlipidemia. Since then, several epidemiological studies have associated consumption of whole grain cereals with decreased risk of chronic diseases such as cardiovascular disease [2], cancer [3] diabetes [4] and obesity [5]. To date, these benefits have been mostly attributed to the content of dietary fiber, essential fatty acids, vitamins and antioxidant phytochemicals including several phenolic compounds in these cereals [6,7]. Different cereals are prevalent in the diets of different communities in the world. Rice is an important cereal amongst Asians, whereas a Western diet is based on wheat, and maize is the major starch food in many parts of Africa. The increasing use of wheat, rice and maize in the human diet has led to a decrease in the consumption of oat and especially barley.

However, both these cereals are still intensively cultivated for other purposes. For example, according to Eurostat statistics, among the cereals cultivated in Europe, wheat is the largest crop, followed by barley, with oats in the third place [8]. The largest producers of oats in 2014 were Poland, Finland and UK, whereas the largest producers of barley were France, Germany and Spain. Data relative to the total production, area under production and average yield of oats and barley in Europe in 2014 has been summarised in Table 1.

**Table 1:** Production of oats and barley in Europe in 2014.

	Total Production’000 tons	Area’000 hectares	Average Yield tons/hectare
Oats	8083	2536	3.1
Barley	67,050	12,398	5.4

Barley is usually classified as spring and winter types, two-rowed or six-rowed (depending on the number of rows of seeds on each spike) and hulled or hulless (by presence or absence of hull tightly adhering to the grain) [9]. Based on the grain composition, barley is further classified as normal, waxy or high amylose starch types, high β-glucan and proanthocyanidin free types. Hulless barley, owing to the absence of hull, requires minimal processing and retains most of the germ and endosperm which is occasionally lost in the process of pearling or dehulling. Therefore, it is more suited for human consumption as the whole grain can be directly used to form a meal or milled to flour. Hulless barley on the other hand is preferred for malting and brewing because of contribution of the hull to beer flavour and as a filtering aid during brewing. Waxy barley genotypes not only deliver unique physical properties to food products but also contain higher contents of protein and β-glucan than genotypes with regular starch composition. Oat can be classified into husked or naked type (depending on the presence or absence of a thick fibrous husk) [10]. Husked oat is suitable for feeding to ruminants. However, unlike husked oat, naked oat has increased metabolizable energy content and low amount of fiber thus making it more suitable for human consumption and as a feed for monogastric animals.

About 14% of the oats produced in Europe are used for the purpose of human consumption [8]. Oats are generally regarded as a minor cereal crop when considered in terms of grain produced annually, or areas sown for production. Oats have been mainly used as animal feed crop, but only in the 19th century it won acceptance as a part of the human diet [11]. Today, oat can be found in various food products such as breakfast cereals, beverages, bread and infant foods [12,13]. In Europe, approximately two-thirds of cultivated barley has been used for animal feed, one third for malting, and approximately <1% for food directly [8]. Barley is primarily used as an animal feed, and as grain for malting and brewing in the production of beer and whiskey. However, recently barley is being used to some extent in their whole, flaked and ground form in breakfast cereals, stews, soups, porridge, flat breads *etc.* [14,15].

Oat (*Avena sativa*) and barley (*Hordeum vulgare*), although consumed in considerably lower quantities than rice and wheat, have the advantage that they are normally consumed as whole grain cereals and considered as a ‘health food’ for humans. Whole grains or foods made from whole grains include the outer bran, the endosperm in the middle and the inner germ, in contrast to the refined grains, in which the bran and germ of the grains are removed during the milling process. Whole grain oats and barley are also good potential sources of fiber, vitamins, minerals and bioactive compounds such as phenolics, carotenoids, vitamin E, phytic acid, β-glucan and sterols [7,16]. Furthermore, oats are one of the few grains that have been recommended in the diet of patients suffering from coeliac disease, since they do not contain gluten [17]. The bioactive compounds present in whole grains may provide desirable health benefits beyond basic nutrition, such as a reduced risk of chronic diseases such as coronary heart diseases [18], type 2 diabetes [19], and cancer [3]. For example, Tighe *et al.* [20] reported 6 and 3 mm Hg reductions in systolic blood pressure and pulse pressure, respectively, among middle aged healthy individuals consuming three servings of whole grain food/day compared with individuals consuming refined grains. This observed decrease in systolic blood pressure is estimated to lower the risk of coronary artery disease and stroke by ≥15% and ≥25% respectively [20]. The health benefits of diets rich in whole grains may be a consequence of the additive effects of nutritional and biologically active compounds present in them [21]. It is clear however that the beneficial properties of oats and barley have attracted much attention from researchers recently. The food industry is also keen on increasing the usage of these cereals as food ingredients and therefore more research is merited in this area.

The bioactive components of oats and barley can be broadly classified into polar and intermediately polar compounds such as β-glucan, phenolic acids, polyphenols, and non-polar compounds such as tocols. A wide variety of techniques have been used over the period of years, for the extraction of these phytochemicals from these cereals. The aim of this article is to provide an insight on the important bioactive compounds that have been extracted from oats and barley. The review discusses the various extraction and characterisation techniques that have been used for identification of these bioactives. The potential shown by novel processing technologies like ultrasound assisted extraction and pressurized liquid extraction in efficiently extracting various bioactives from oats, barley and other related cereals has also been discussed.

## 2. β-Glucan

**Figure 1:**
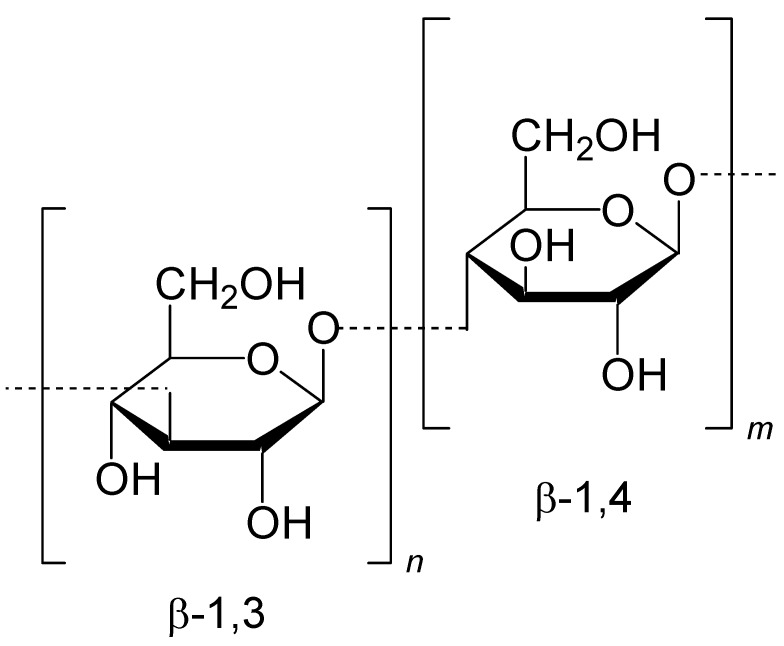
Chemical structure showing the β-1,3 and β-1,4 bonds of β-glucan.

Research on oats and barley has intensified arising from the findings that oat and barley bran possess serum cholesterol lowering and hypoglycaemic effects [22,23]. This property has been largely attributed to the soluble fiber fraction of these cereals, in particular to the (1-3, 1-4) β-d-glucan (β-glucan, Figure 1) component. Barley and oat β-glucans, together with other non-starch polysaccharides, occur in the walls of the endosperm cells which enclose starch, matrix protein and lipid reserves of the grain. The extraction of β-glucans usually requires inactivation of endogenous enzymes (β-glucanases and amylases), as leaving these enzymes active may degrade the β-glucan resulting in a low molecular weight β-glucan product. Preserving the molecular weight (MW) of β-glucan is an important factor, as it determines its physicochemical properties such as viscosity, which in turn determines the cholesterol-lowering property of β-glucan [24].

Wood *et al.* [25] were amongst the first authors to investigate factors affecting β-glucan extraction efficiency. They used a dehulled oat variety ‘Hinoat’ to assess the effects of particle size, ionic strength, temperature, pH and obtained an oat β-glucan gum fraction by extraction of 75% ethanol refluxed oat bran, with a sodium carbonate solution at pH 10. Refluxing with ethanol was performed for achieving inactivation of the endogenous β-glucanases. This simple procedure yielded a high viscosity β-glucan gum with 78% purity. Several other extractants for the recovery of β-glucan from hulless barley, waxy hulless barley and commercial oat bran were investigated by Bhatty [26]. The authors reported that the highest enrichment of β-glucan from barley and oats could be obtained by a single extraction using 4% of 1 M sodium hydroxide (NaOH) at room temperature. The purified high viscosity gums contained 72%–81% β-glucan plus pentosans from barley brans and 84% from oat brans. The β-glucan yield or the % total recovered from barley bran and oat bran were 81% and 61% respectively. Later, the same approach was used by Bhatty [27] for pilot scale extraction. Although ethanol deactivation and extraction at higher pH with NaOH have been found to yield a high viscosity β-glucan gum, these processes are accompanied with a decrease in yield and an added risk of degradation of the β-glucan polymer [28]. Pre-treatment processes such as boiling and ethanol reflux have also been shown to increase starch contamination of the extract, because of its gelatinisation at temperatures around 63 °C. Thereby, reducing the starch content of the extract from refluxed flour, using thermostable α-amylase resulted in significant improvement in purity of the gums [28]. Recently, an enzymatic procedure has been used to obtain β-glucan from Greek barley cultivars wherein, the removal of starch and proteins was achieved enzymatically using thermostable amylase and pancreatin respectively, followed by precipitation of β-glucans using 37% ammonium sulphate [29]. The purity of β-glucan using this procedure was as high as 93%, with some small amount of protein contamination. The MW of β-glucan of these samples varied from 0.45 × 10^6^ to 1.32 × 10^6^ g/mol.

Various research studies have shown that temperature, pH and pH-temperature interaction of the extraction process are important factors in β-glucan recovery and functionality [28,30,31,32]. As temperature increases beyond 60 °C, the starch contamination of the extract increases due to gelatinisation of starch. Dawkins and Nnanna [31] found that pH/temperature treatment combinations of 9.2/50 °C or 10.5/50 °C/55 °C produced oat gum with little or no starch contamination. Later, Temelli [30] recommended a pH/ temperature combination of 7.0/55 °C for maximising the β-glucan content, gum yield, emulsion stability and viscosity. The ethanol refluxing step was not employed for this extraction, based on the hypothesis that this step might not be required if extraction was carried out in acidic or alkaline conditions, wherein the enzymes were inactivated during the process itself. This hypothesis was proved when the viscosity of the β-glucan gum was found to increase with an increase in pH of the extract. In 2011, a Taguchi experimental design was used to find an optimal combination of factors that maximise the extraction yield from waxy barley cultivars [32]. At pH close to neutrality and temperature of 55 °C, an extraction yield of (73.4 ± 1.2)% was obtained at the optimal conditions of particle size, 100 μm; solvent:flour ratio of 5; stirring rate, 1000 rpm and extraction time, 3 h. However, molecular weight (MW) of β-glucan or its viscosity was not taken into account in the aforementioned study. Recently Gangopadhyay *et al.* [33] obtained similar results on using response surface methodology for optimising extraction of high amount and high MW β-glucan from Irish hulled barley cultivars. Size exclusion chromatography was used for determination of MW of β-glucan. Water used as the extraction solvent at a temperature of 55.7 °C and pH 6.6 were found to be optimal for delivering a high yield (81.5%) and high MW (351.6 kDa) β-glucan product from barley [33]. A technique that has been commercially accepted for making β-glucan from barley flour involves extraction with water followed by freezing the extract [34]. The frozen extract is thawed and the fibrous material is recovered from it by filtration and further washed with water and ethanol before being dried. This β-glucan product known commercially as Glucagel^®^ is a high purity (75% β-glucan) product with MW varying from 62 kDa to 560 kDa depending on the temperature and time used for extraction.

Several techniques have been employed for analysis of the oligosaccharides from enzyme-hydrolysed barley β-glucan. The earliest technique used was paper chromatography [35], which had the disadvantage of being extremely time consuming taking up to 72 h. Oligosaccharides released from the β-glucans of oat products, barley, wheat and rye have been analyzed by the use of HPLC, but this technique could not adequately resolve the oligosaccharides with a higher degree of polymerisation (DP) [36]. Derivatization using methylation, of oligosaccharides released by lichenase hydrolysis from barley β-glucan, improved the HPLC resolution of the oligosaccharides with higher degree of polymerisation [37]. Wood *et al.* [38] successfully applied high-performance anion-exchange chromatography with pulsed amperometric detection (HPAEC-PAD) for analysis of oligosaccharides from barley β-glucan, with DP of 5–19. However, the quantification of oligosaccharides by HPAEC-PAD was limited to the knowledge of weight response factors.

Jiang and Vasantham [39] were the first to investigate the use of MALDI-MS for the qualitative and quantitative analyses of oligosaccharides, of lichenase-hydrolyzed water soluble β-glucan from barley. The authors concluded that MALDI-MS provides a rapid and sensitive means for the analysis of the oligosaccharides and requires only 1/12 of the sample concentration of that used by HPLC or HPAEC-PAD. A standard addition method was employed to determine both the relative and absolute amounts of each oligosaccharide using the weight concentration relationship. Oligosaccharides from DP 3 to 12 could be detected and quantified by this method. Trisaccharide (DP 3) and tetrasaccharide (DP 4) chain units were indicated to be the main building blocks of water soluble barley β-glucan, accounting for about 95% of the whole polymer, with the molar ratio of tri- to tetrasaccharides ranging from 2.3 to 2.8.

Nuclear magnetic resonance (NMR) spectroscopy is a method that has been widely used for the primary and secondary structural determination of β-glucan. Johansson *et al.* [40] used two-dimensional correlation NMR for structural characterization of water soluble β-glucan. They showed that the glucose units are joined with 1,3- and 1,4-linkages only, and that no other linkages exist. Later Johansson *et al.* [41] also studied structural differences between oats and barley, and soluble and insoluble β-glucan using FT-IR, ^1^H-NMR and solid state ^13^C-NMR. They showed differences in the ratio of oligosaccharides with degree of polymerisation 3 and 4 (DP3:DP4) in oats and barley. The ratio of DP3:DP4 was higher for barley than for oats, indicating a higher number of cellotriose segments in barley. Ryu *et al.* [42] linked the structural differences between the barley and oat β-glucan to their rheological and thermal characteristics. It was suggested that oat with a lower DP3:DP4 and thus higher cellotetraosyl units would have improved solution viscosity and gel formation characteristics than barley.

## 3. Phenolic Compounds

In human cells, radicals are formed from normal metabolism as well as environmental radiation. These radicals can cause changes in DNA, which may lead to production of cancerous cells or diseases like atherosclerosis. They may cause cross linking between molecules of fat and protein leading to wrinkling of skin. Free radicals are also known to oxidize LDL-cholesterol, which may contribute to the development of heart diseases and strokes. Thus, free radicals are thought to be key casual factors in many of the chronic diseases. The human body has a natural defence system against these reactions but dietary antioxidants also contribute to the body’s defence.

Unfortunately, the contribution of grain consumption to antioxidant defences has been largely ignored, in spite of its role as a staple dietary component for most of the world’s population. Health promoting effects of whole grain cereals have been partly related to their content of polyphenols, which are often enriched in the outer part of the cereal grain [43]. Oats and barley have a wide spectrum of phytochemicals which may be antioxidants themselves or act as a source of antioxidant metabolites that are formed in the colon. Recent evidence suggests that the complex mixture of bioactive components in wholegrain cereals may be more beneficial to health than individual isolated components [21]. The main sources of antioxidants in oats and barley are the various phenolic compounds (phenolic acids, flavonoids), tocols (vitamin E) and phytic acid.

**Figure 2:**
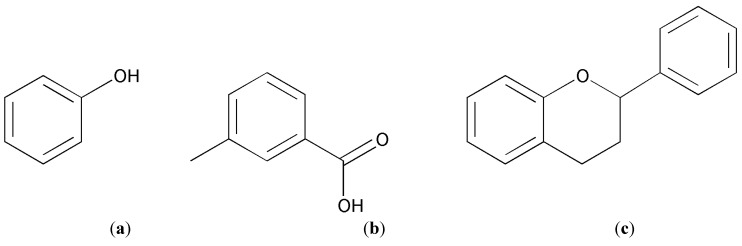
Structures of common phenolic compounds (**a**) phenol; (**b**) phenolic acid; (**c**) flavonoid.

Phenolic compounds are made of one or more aromatic rings and contain one or more hydroxyl groups. They vary greatly in complexity from very simple to highly polymerised phenols. They are generally categorized as phenolic acids, flavonoids, stilbenes, coumarins and tannins [21] (Figure 2). Phenolics are products of secondary metabolism in plants, and play important roles in their defense mechanisms against pathogens and parasites, and some of them even contribute to the colour of plants [44]. In addition to their role in plants as defence compounds, phenolic compounds in our diet contribute to protection against chronic diseases. Various cell culture and animal model studies have demonstrated the ability of polyphenols to inhibit the development of cancer. Various mechanisms such as antioxidant activity involving reactive oxygen species, enzyme modulation, gene expression, and apoptosis have been suggested, by which polyphenols exhibit their anti-carcinogenic activity [45]. Polyphenols also show protective effects against heart diseases possibly by preventing the oxidation of LDL-cholesterol into an atherogenic form, which is a kind of fatty material that deposits on the inner wall of arteries. The concentration of phenolic compounds in whole grain cereals is influenced by the grain type, variety and part of grain sampled. The most common phenolic compounds found in whole grain cereals are phenolic acids and flavonoids. Avenanthramides (AVs) are another class of polyphenols that have been specifically reported in oat. Phenolic compounds are mostly located in the oat bran as compared to the oat groat, whereas AVs are more uniformly distributed throughout the groat [46]. The following section will discuss in detail factors which influence the recovery of polyphenol groups from cereal grains.

### 3.1. Phenolic Acids

Phenolic acids are hydroxylated derivatives of either benzoic acid or cinnamic acid. Hence they are divided into two major groups:hydroxybenzoic acids and hydroxycinnamic acids. Hydroxycinnamic acids are more common than hydroxybenzoic acids and chiefly consist of *p*-coumaric, caffeic, ferulic and sinapic acids. Hydroxybenzoic acid derivatives include *p*-hydroxybenzoic, protocatechuic, vanillic, syringic and gallic acids.

Phenolic acids occur in cereals in both free and bound forms. Free phenolic acids are found in outer layer of the pericarp whereas bound phenolic acids are ester-linked to cell walls and require acid, base or enzymatic hydrolysis to release them from the cell matrix [47]. Durkee and Thivierge [48] reported low levels of free ferulic, vanillic, sinapic and *p*-hydroxybenzoic acids in 70% ethanol solid liquid extracts from oat grains. However the bulk of ferulic, sinapic, *p*-coumaric and hydroxybenzoic acids were derived from soluble or insoluble bound forms *i.e.*, released by alkaline hydrolysis or β glucosidase. These results led the authors to believe that these phenolic acids mainly existed as soluble esters and insoluble esters with polysaccharides, proteins or cell wall. They also quantified the amount of ferulic acid in oat grain to be 300mg/kg, the majority of it being in bound forms.

The first quantitative data on the various phenolic acids in oats, other than ferulic acid, was reported by Sosulski *et al.* [47]. The authors extracted phenolic acids from oat flour and separated them into three fractions: free, soluble esters and insoluble bound. The flour was extracted with methanol:acetone:water (7:7:6) at room temperature. After centrifugation, the combined supernatants were analyzed for free phenolic acids and soluble-bound phenolic acids and the residue for insoluble-bound phenolics. The free phenolic acids were then extracted with diethyl ether:ethyl acetate (1:1). The soluble esters remaining in the aqueous extract were hydrolyzed with 4 N NaOH and extracted with ether:ethyl acetate. Insoluble-bound phenolics were obtained by hydrolysis of the residue from the methanol/acetone extraction using 2 N NaOH, followed by acidification, centrifugation and extraction with ether:ethyl acetate. Quantification of the phenolic acids was done by Gas Liquid Chromatography (GLC) and Gas Chromatography-Mass spectrometry (GC-MS). The amount of total free phenolic acid was 8.7 mg/kg, whereas soluble phenolic esters and insoluble phenolic acids totalled 20.6 mg/kg and 57.7 mg/kg, respectively. Ferulic acid was detected in all the three fractions, but largely dominated the insoluble bound residue. Other phenolic acids such as *p*-hydroxybenzoic, coumaric, vanillic, syringic acids were only found in the other two fractions. The above studies suggested ferulic acid to be the most abundant hydroxycinnamic acid found in oat grains most of which occurs in bound form as oxidatively coupled dimers that are esterified to the arabinoxylans in the cereal cell wall [49]. Three new phenolic acids have been identified by a Canadian group in oat groats and hulls, namely avenalumic acid and its 3ʹ-hydroxy and 3ʹ-methoxy derivatives [50]. These acids are ethylenic homologues of the E-isomers of *p*-coumaric, caffeic and ferulic acids. In the oat grain they occur in bound form, covalently linked to the amine group of hydroxy-substituted amino-benzoic acids. The structures of all these acids were determined by mass spectral and nuclear magnetic resonance spectral analysis.

Bonoli *et al.* [51] compared the extraction capacity of several solvent mixtures such as methanol/water, ethanol/water and acetone/water and also investigated the use of pressurized liquid extraction (PLE) for extracting free phenols from barley flour. The extraction yields were evaluated using spectrophotometric indices (absorption at 280 nm, 320 nm and 370 nm or and total phenolic content). Acetone/water (4/1 *v*/*v*) and ethanol/water (4/1 *v*/*v*) led to higher extraction yields of free phenols whereas a less satisfactory recovery was obtained with use of PLE. In the same study, a prolonged 20 h alkaline hydrolysis was demonstrated to be efficient for extracting hydroxycinnamic acids, while acid hydrolysis allowed higher yield of the generic phenols. Thus, information on bound phenolics could be obtained from both hydrolysis methods. A similar comparative study between different solvents for the extraction of free and bound phenolic compounds in oats was also carried out [52]. The extraction yield of each method was evaluated by correlating various spectrophotometric indices with HPLC-MS and antioxidant activities of the oat extracts. The results demonstrated that 4/1 methanol/water (*v*/*v*) extracts and alkaline hydrolysis extracts showed the highest content of free and bound polar compounds in oats, respectively.

In another quantitative analysis, the level of bound phenolic acids in barley flour (wholemeal and air-classified fractions) was determined [53]. Acid hydrolysis was carried out for discharging the bound phenols followed by their characterization and quantification by spectrophotometry and reversed-phase high-performance liquid chromatography (RP-HPLC) coupled with UV-diode array and mass spectrometry (UV-DAD-MS) detectors. HPLC-MS in both positive- and negative-ion mode helped in identification and quantitation of the main peaks of 12 different hydroxycinnamic acids, which were found to be the main bound polyphenols in the barley flours. In congruence with other studies, this analyses too demonstrated ferulic acid to be the most abundant hydroxycinnamic acid, constituting 89%–93% of the total phenolics. The study carried out by Holtekjølen *et al.* [54] was on similar lines, where HPLC-DAD-MS analysis detected total bound phenolic acids ranging from 604 μg/g to 1346 μg/g of barley flour, with the monomeric ferulic acid being the most abundant, followed by coumaric acid. In addition, observed were diferulic acid and triferulic acid in the bound fraction.

Yu *et al.* [55] were the first to investigate enzymatic hydrolysis for the release of bound polyphenols from barley. They investigated phenolic acids from 30 barley varieties using HPLC-UV detector following four different pre-treatments: (a) hot water extraction for free phenols (b) acid hydrolysis (c) acid hydrolysis followed by α-amylase treatment and (d) acid hydrolysis followed by α-amylase and cellulase treatment for bound phenols. It was inferred that a combination of sequential acid, α-amylase, and cellulase hydrolysis treatments yielded the highest concentration of phenolic acids than acid hydrolysis alone, indicating that most phenolic acids in barley are bound to other grain components (for example, starch, cellulose, β-glucan, pentosans, and others) by complex bonds. The quantitative data differed from the other studies in that p-hydroxybenzoic acid was found to be the major phenolic acid in all the barley varieties followed by ferulic acid.

### 3.2. Avenanthramides

**Figure 3:**
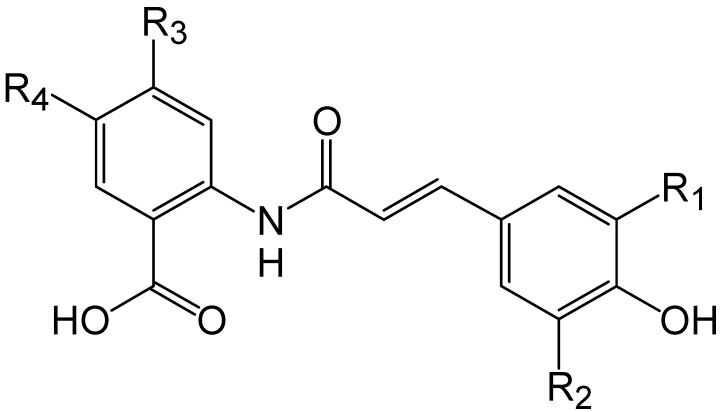
Structures of the derivatives of avenanthramides.

Avenanthramides (AVs) are a group of hydroxycinnamoyl anthranilate alkaloids that were first detected and characterized by Collins [56]. They have been exclusively reported in oat groats and hulls, and partially represent the alcohol soluble phenolic antioxidants found in the oat kernel [57,58,59]. AVs constitute about 0.2–0.8 mg per gram of bran-rich milled fractions of oats [60] and consist of amide conjugates of anthranilic acid or its hydroxylated derivatives and hydroxycinnamic acids. Six AVs derived from anthranilic acid (1), 5-hydroxyanthranilic acid (2), or 5-hydroxy-4-methoxyanthranilic acid (3) and *p*-coumaric (p), caffeic (c) or ferulic (f) acid, namely, 1p, 1f, 2p, 2c, 2f, and 3f, have been isolated and identified in oat grains [56,57,58] and these differ with regard to the substituents on the cinnamic and anthranilic acid rings (Figure 3).

AVs have been isolated from grains for structure elucidation purposes using a variety of methods. Collins [56] used ion exchange chromatography for anionic fractionation of the extract obtained from oat groat and hull using 80% methanol to separate the free phenolics from the complex mixture of avenanthramides. Further two-dimensional thin layer chromatography of this group of AVs showed the groat extracts to contain around 25 distinct AVs and the hull extracts to contain 20, out of which 15 were common to both groat and hull preparations. Structures of 10 AVs were elucidated using ^1^H- and ^13^C-NMR, MS and UV techniques and confirmed by chemical synthesis. These AVs were all N-cinnamoylanthranilate alkaloids known as A-E, whereby avenanthramides A, B, C, D, E existed in E configuration and A-1, B-1, C-1, D-1, E-1 existed in Z configuration. In daylight and UV light, the AVs may easily undergo Z-E rearrangement. Dimberg *et al.* [57] separated two avenanthramides, from the 80% ethanolic extract of oats by column chromatography and prep TLC. The authors elucidated structures of the two avenanthramides by ^1^H-NMR and UV spectroscopy, and named them as A1 [*N*-(4ʹ-hydroxy-3ʹ-methoxy-(*E*)-cinnamoyl)-5-hydroxyanthranilic acid] and A2 [*N*-(4ʹ-hydroxy-3ʹ-methoxy-(*E*)-cinnamoyl)-5-hydroxy-4-methoxyanthranilic acid]. The former corresponded to the avenanthramide B as reported by Collins. The latter (A2) was not previously described in literature. It was however present in 10 times lower amounts than that of A1. The quantities of A1 varied from 40–132 μg/g among the 10 cultivars of oats tested. Recently a new series of AVs containing a 4,5-dihydroxyanthranilate moiety were purified using column chromatography and HPLC from oat seeds [61]. The structures of these AVs were elucidated by a LC-MS/MS analysis with multiple reaction monitoring.

**Table:** 

Avenanthramide	Collin’s Nomenclature	R_1_	R_2_	R_3_	R_4_
1p	D	H	H	H	H
1c		H	OH	H	H
1f	E	H	OCH_3_	H	H
2p	A	OH	H	H	H
2c	C	OH	OH	H	H
2f	B	OH	OCH_3_	H	H
3f		OH	OCH_3_	H	OCH_3_

Later in 1996, Dimberg *et al.* [58] studied the effect of variety, storage and heat treatment on the phenolic compounds present in three varieties of oat. Eleven UV-absorbing compounds, three of which were avenanthramides, were identified and quantified from the ethanolic extract of oat groat using HPLC with a diode array detector. They were named as AV1, AV3 and AV4 and corresponded to B, C and A respectively of Collins’ classification. The variety Mustang was reported to have a higher concentration of all the three AVs than the other two varieties, namely Svea and Kapp. Storage had no effect on the levels of AV in the oat varieties, but heat treatment led to a small reduction in the levels of AV1 and AV3. AV1 has been found to be a rather heat stable antioxidant in oat and might play a role in preventing the development of oxidative rancidity in thermally processed oat. AV4 has been found to be more heat labile amongst the three avenanthramides.

### 3.3. Flavonoid

Flavonoids are a large group of polyphenolic compounds with a C6-C3-C6 skeleton. The basic molecular structure of flavonoids consists of an aromatic ring A condensed to a heterocyclic oxygen ring C and attached to a second aromatic ring B (Figure 2c), all of which exhibit various levels of hydroxylation and methoxylation [62]. The various subclasses of flavonoids include flavonols, flavones, flavanols, flavonones, flavans and anthocyanins. Flavonoids found in fruits and vegetables have also been reported in cereals. For example the flavone apigenin, a compound found in celery, is also been reported in barley, sorghum, millet and oat [63]. However cereals have only a small quantity of flavonoids and not much work has been published on these compounds from oats.

Collins and Webster [64] identified the major flavones present in oat flour as apigenin, luteolin and tricin. Flavonols such as quercetin and kaempferol have also been identified in oat and barley. The flavone tricin has recently been reported in the polar fraction of five Romanian oat varieties, extracted using 4/1 (*v*/*v*) methanol/water, 4/1 (*v*/*v*) ethanol/water and 4/1 (*v*/*v*) acetone/water [52]. Reversed phase HPLC DAD coupled to a mass spectrometer was then used for identification and quantification of the phenolics in the polar fraction. In one of the varieties ‘Comun’, tricin was the principal compound, and represented more than 50% of the free polar compound fraction. In another study, flavonoids were extracted from barley and hops [65] using (75:25 *v*/*v*) acetone and subjected to fractionation. As a first fractionating step, the acetone-water extract was saturated with sodium chloride and allowed to separate. The upper liquid phase was mixed with hexane before a second phase separation. The lower liquid phase obtained from this phase separation was termed as the flavonoid extract. Reversed phased HPLC was used for separation and quantitation of the flavonol glycosides and simple flavanol oligomers in the flavonoid extract. However, resolution of polymeric flavanols could not be achieved by this method. Flavonoids belonging to the family of proanthocyanidins have been identified in barley [66]. Proanthocyanidins (PAs) are polymeric flavanols whose building blocks are the monomeric flavanols including catechin and epicatechin. PAs are excellent chelators of iron (Fe^3+^) and inhibit the absorption of non-haem dietary iron through the gut barrier. Thus, they influence the bioavailability of iron in the body and may be effective in treating an iron overload. Five different flavanols have been identified in different barley varieties namely, (2*R*,3*S*)-catechin-7-*O*-β-d-glucopyranoside, prodelphinidin B3, procyanidin B3, catechin and procyanidin B1, using ^1^H-NMR and quadrupole time-of-flight mass spectrometry [66]. Catechin was found to be present at the highest concentrations amongst those analyzed, in all varieties. Principal component analysis indicated a strong correlation between the concentrations of catechin and prodelphinidin B3.

## 4. Lipids

Apart from their soluble fiber and phenolic content, barley and oat are also a source of lipids/oil. The lipids in these cereals are mainly concentrated in the germ of the grain. The lipid fraction of cereal grains determines to a greater extent its energy content and has a significant impact on its nutritional quality via the fatty acid content. In oat, lipids are also implicated in its flavour/off-flavour attributes. According to Zhou *et al.* [67], oat presents the highest oil content between 2% and 12% among other cereal grains. The neutral lipids in oat are triacyglycerols, which account for 50%–60% of the oat lipids. Oat oil is also rich in polar lipids such as phospholipids and glycolipids [67]. Barley contains about 2%–4% lipids, in which the average values for neutral lipids, glycolipids and phospholipids are 70%, 9% and 20% respectively [68].

### 4.1. Phospholipids

The phospholipid content of barley and oat has been scarcely studied despite the role of phospholipids in growth of all the body cells, and their role in the structure and function of cell membranes. The amount of phospholipids in oat is about 5%–26% of the total lipids. The very first of the studies on lipids in barley was conducted by Parsons and Price [68], where fractionation of the phospholipids by two-dimensional thin layer chromatography (TLC) followed by their identification using gas chromatography (GC), revealed phosphophotidylcholine and phosphotidylethanolamine to be present in highest amounts. The fatty acid linoleic acid (18:2) was found to be predominant in most of the fractions followed by palmitic acid (16:2). Sequential extraction of oats or groats with hexane, followed by solvents of increasing polarity provides a crude fractionation of the lipids into nonpolar (neutral or free) and polar (bound) lipids. Supercritical fluid extraction (SFE) has provided a novel alternative to lipid recovery and fractionation from oat [69]. SFE (particularly with supercritical carbon dioxide) provides a more homogeneous neutral lipid extract than conventional extraction with hexane. To investigate the phospholipid fraction of oat lipids, Barnes [70] extracted all lipids using 1:1 chloroform:methanol. HPLC combined with evaporative light scattering detector was used for quantification of the phospholipids, where phosphatidylethanolamine was found to be the most abundant in all oat samples. Further HPLC combined with mass spectrometry was used for determination of the molecular species of each individual phospholipid class. Using preparative TLC and later GC, linoleic acid (C18:2) was demonstrated as the main fatty acid of the phospholipid fraction.

### 4.2. Phytosterols

The unsaponifiable lipid fraction of plant based foods is a potential source of phytosterols that are integral natural components of plant cell membranes. While the levels of phytosterols in cereals are moderate, high levels of them have been detected in the pearling fines (outer kernels) of barley [71]. More recently, a special milling process (Fitzpatrick Comminuting mill) was used to produce a germ enriched fraction, rich in phytosterols from barley [72]. The potential use of pearling/milling as a process to generate fractions with high levels of phytosterols was proposed in this study. In another study the level of sterols in different cereals including oat and barley was compared [73]. For extraction of the sterols, the samples of milled grains were first acid hydrolysed. To free the esterified phytosterols and to remove the glycerol lipids, the extracts were then saponified using potassium hydroxide (KOH) and absolute ethanol. A liquid-liquid partitioning was then carried out using hexane and water to extract the unsaponifiables in the organic phase. The extracted sterols were then derivatised (silylated) and analyzed using gas chromatography with a flame ionisation detector (GC-FID). The total sterol content of barley (76.1 mg/100 g) was much higher than in oats (44.7 mg/100 g). The type of sterols found in barley and oats were similar, with sitosterol being the most prominent, followed by campesterol, stigmasterol and brassicasterol.

Phytosterols have been widely studied because of their ability to control serum cholesterol levels and thus protect against cardiovascular diseases. While they lower the levels of LDL-cholesterol, levels of ‘good’ HDL-cholesterol and triacyglycerols remain unaffected [74]. The beneficial health effects of plant sterols have led to the development of functional foods enriched in sterols. The possibility of use of sterols as an adjunctive therapy to cholesterol reducing pharmaceuticals has also been proposed recently [75].

### 4.3. Tocols

**Figure 4:**
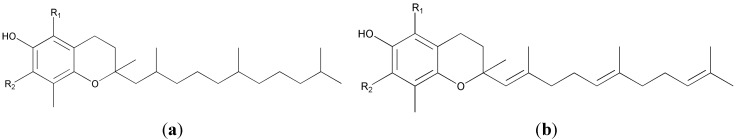
Structures of tocols. (**a**) Tocopherols; (**b**) Tocotrienols.

Vitamin E is the generic name for the lipid soluble compounds, known as tocols, that exist in cereal grains and other sources. Tocols are mainly located in the germ of cereals from which Vitamin E rich oils are extracted. They are lipophilic and are intimately associated with lipid components of the sample matrix. Sample preparation procedures for analysis involve either solvent extraction or saponification using alkaline hydrolysis. Tocols include tocopherols (TP) and tocotrienols (TT) (Figure 4). There are 4 homologues for tocopherol, α, β, γ and δ, which differ in the number and position of methyl groups on the chroman ring structure [76]. A similar series exists for tocotrienols. The chemical structures of the tocols determine their vitamin E activity. Different isomers of tocols exhibit vitamin E activity in the following order αTP > βTP > αTT > γTP > βTT > δTP [77]. Although α-TP has the highest vitamin E activity, αTT has been found to possess 40–60 times higher antioxidant activity than αTP due to the higher recycling efficiency of its chromanols from chromanoxyl radicals and its more uniform distribution in the membrane bilayer. The main source of tocols is vegetable oils, but substantial amounts have been found in most cereal grains including barley, oats, wheat, rye, rice.

**Table:** 

	R_1_	R_2_
α	CH_3_	CH_3_
β	CH_3_	H
γ	H	CH_3_
δ	H	H

In barley and oat, all eight tocol isomers have been reported [70,78]. In a survey of 12 oats and 30 barley genotypes, grown at three different locations in the USA, the tocol concentration ranged from 19 mg/kg to 30 mg/kg for oat and 42 mg/kg to 80 mg/kg for barley [79]. The concentrations were significantly affected by genotype and location in oats but not in barley. In this study, the tocols were first extracted from the ground grain with methanol. Following evaporation of the solvent, the tocols were extracted with hexane. They were then separated and quantified using HPLC with a fluorescence monitor that employed an excitation wavelength of 295 nm and emission wavelength of 330 nm. The oat samples predominantly contained α-tocotrienol and α-tocopherol, with lesser amounts of β-isomers, γ-tocopherol, and δ-tocotrienol. In barley too, α-tocotrienol was the major fraction followed by α-tocopherol. However, barley also had substantial quantities of β- and γ-tocotrienols. Typically, tocols have been extracted from dried oat samples using methanol. However, saponification has been found to be less time consuming with the ability of increasing the yield of tocols up to ~25% [76]. Panfili *et al.* [80] used hot saponification using potassium hydroxide, sodium chloride and ethanol as the elective method for tocol recoveries from oat. Hot saponification resulted in significantly higher tocol recoveries as compared to the methanol extraction. After extraction, the authors used normal phase HPLC instead of reversed phase for the simultaneous detection and quantification of tocols. Normal phase HPLC allowed an efficient separation of the β- and γ- isomers of tocopherols and tocotrienols, which is otherwise difficult to achieve on reversed phase columns.

The distribution of tocol isomers in the kernel is uneven. The germ of a grain is the location for most of the α- and γ-tocopherol, whereas almost all the tocotrienols are located in the bran of the endosperm and absent from the germ [81]. Tocols have been found to be stable in unprocessed groats for a period of 7 months at room temperature. However, they undergo degradation in all the processed products.

## 5. Proteins and Bioactive Peptides

Dietary proteins from various food sources have long been studied for the presence of latent bioactive peptide sequences within their primary structure, which may have the ability to exert a physiological response *in vivo*. A large number of these biofunctional peptides have been isolated from food proteins including anti-cancer, anti-inflammatory, immunomodulatory, muscle-stimulating and angiotensin converting enzyme (ACE) inhibitory peptides. Cereal grains though high in carbohydrates, also contain a substantial amount of proteins, thus possessing the potential to provide bioactive peptides in the diet. Protein content in cereals ranges from 10%–15% of the dry grain. A big share of this comes from storage proteins. The seed storage proteins in cereals are classified according to their solubility in water (albumins), dilute saline (globulins), alcohol/water mixtures (prolamins) and dilute acid (glutelins) [82]. The major storage proteins in barley are prolamins, specifically hordeins, while the major storage proteins in oats are globulins known as avenalins [83]. Avenalins make up to 80% of the total oat protein, whereas prolamins (avenins), account for about 15%. Oat proteins are also considered safe and can be tolerated by patients with gluten intolerance/coeliac disease [17].

Sequential extraction procedures using different solvents to achieve complete and separate extraction of the various protein fractions in oat and barley, followed by their quantitation has been conducted [84,85]. For example, Howard *et al.* [85] extracted the different protein fractions from ground barley flour by sequentially using three different solvents. The salt extractable proteins (albumins and globulins) were first extracted using potassium phosphate (pH 8.0) containing dithiotrietol at room temperature. Similarly, the alcohol extractable proteins (hordeins) were extracted with 55% 2-propanol, 1% acetic acid and 2% mercaptoethanol at 60 °C. The residual proteins glutelins were extracted with 0.05 M sodium borate (pH 10) containing 2% 2-mercaptoethanol and 1% sodium dodecyl sulfate. The use of detergent with a reducing agent (like sodium dodecyl sulphate) was to increase the extraction efficiency of the protein fractions. However, this procedure involves the concern of reagent contamination in the final protein isolate. In another study, Wang *et al.* [86] used the process of pearling to separate the barley outer layers, pearling flour (PF), from the endosperm, pearling grain flour (PGF), so that the barley albumin and globulin would be enriched in PF, while hordein and glutelin would be enriched in PGF. PF was then treated with sodium chloride (NaCl) solution and alkaline solution (pH 9–11.5) adjusted with 0.5 M NaOH) in two separate steps for cytoplasmic protein extraction (albumin and globulin). The PGF was treated with ethanol solution (55%–75%, *v*/*v*) to isolate the hordein fraction, and the residue was treated with alkaline solution (pH 9–11.5) to extract glutelin. The protein content and the functional properties including the oil-binding property, foaming capacity and emulsifying stability of the protein fractions were determined, however, their bioactivity or the presence of bioactive peptides in these protein fractions were not studied.

The storage proteins in oat and barley have recently been sequenced and the possibility of presence of bioactive peptides in them has been determined by *in silico* methods using the BIOPEP database [87]. Sequences with various biological activities such as Angiotensin-I converting enzyme (ACE) inhibitors, prolyl endopeptidase inhibitors, renin inhibitors have been identified in the cereal protein sequences. In the last decade, search for bioactive peptides especially ACE inhibitory peptides from various food protein sources, has gained momentum. ACE is a multifunctional enzyme in the renin-angiotensin system that is responsible for an increase in hypertension, which is the leading cause of cardiovascular diseases and deaths in the world. Bioactive peptides derived from food may play a part in prevention and control of these diseases along with the advantage of having less side effects compared to the synthetic drugs. Recently, the potential of producing potent ACE inhibitory peptides from hydrolysis of oat protein isolates was assessed *in silico* and confirmed *in vitro* in a study [88]. Application of the “enzyme action” tool in the BIOPEP database on three oat protein sequences, indicated that many enzymes including chymotrypsin C, ficin, pancreatic elastase, papain and thermolysin displayed the theoretical ability to release peptides with “antihypertensive” activity. Amongst these, thermolysin was predicted to yield a much greater number of these peptides. This was then verified by conducting experiments *in vitro* where the ACE-inhibitory activity of hydrolysate produced with thermolysin was much higher with an IC_50_ below 20 μM.

Detection and characterisation of ACE inhibitory peptides from barley protein hydrolysates has not yet been conducted. However, lunasin a novel 4.8 kDa cancer preventive seed-peptide, has been reported to be prevalent in barley [89]. Lunasin enriched barley (LEB) was obtained by extracting barley flour with phosphate-buffered saline supplemented with protease inhibitor cocktail for 48 h at 4 °C. Lunasin was further purified from the LEB using ion exchange and immunoaffinity column chromatography followed by its identification using MALDI peptide mass mapping. The purified lunasin was demonstrated to inhibit colony formation in ras-transfected mouse fibroblast cells, just as effectively as synthetic lunasin. Moreover, the purified fractions were also seen to inhibit histone acetylation in mouse fibroblast and human breast cells. In another report, the bioavailability of lunasin *in vivo* has been studied [90]. Oral administration is an important feature of an ideal cancer preventive agent and hence the LEB was orally fed to rats, to establish if barley lunasin survives digestion and remains intact. The liver and kidney of rats fed with LEB showed the presence of lunasin in Western Blot.

Table 2 outlines a brief overview of the extraction techniques, associated health benefits and commercial availability of the various bioactive components present in oat and barley.

**Table 2:** Phytochemicals present in barley and oats, methods used for extracting them and their reported bioactivities.

Phytochemical	Source	Extraction Solvent/Technique Used	Bioactivity	Commercial Availability
β-glucan	Barley	4% 1 M NaOH [27]; Water at 55 °C and pH 7.0 [32]; Pressurized hot water extraction at 155 °C, 18 min and 50 bar [91]	Lowers cholesterol [92] reducing the chances of coronary and ischemic heart disease. Lowers glycaemic index and postprandial glucose levels [93].	Barley β-glucan is available as Glucagel^®^, PromOat^®^, Glucan 300^®^
	Oat	Ethanol reflux followed by extraction with a sodium carbonate solution at pH 10 [26].		
Phenolic acids (PA)	Barley	Free phenolics—Acetone/water (4/1) [51,94]; ethanol/water (4/1) [51]; pressurized liquid extraction [61]. Bound phenolics—20 h prolonged alkaline hydrolysis [51]; Acid hydrolysis [53]; Sequential acid, α amylase and cellulose hydrolysis [55]	PA act as antioxidants and protect against the destructive activity of free radicals. They reduce the risk of chronic age-related diseases such as cardiovascular diseases, diabetes and ageing, by reducing oxidative stress [95]. PA bound to dietary fiber may prevent cancer of the colon [96].	Green tea extracts rich in phenolic acids are available as Bulk Powders^®^ and Super Green Tea Diet^®^
	Oat	Free phenolics—Methanol [97], Methanol/water (4/1) [52]; Acetone/water (1/1) [95]; 80% ethanol [46,57]; Bound phenolics—Alkaline hydrolysis [52], acid hydrolysis [52,53]		
Avenanthramides (AV)	Oat	80% methanol [56,98]; 80% ethanol [57,58].	AV are bioavailable and act as antioxidants by inhibiting LDL oxidation in synergy with Vitamin C [99]. They demonstrate anti-allergic activities.	
Flavonoid	Barley	40% ethanol [100]	Proanthocyanidins have ferrous chelating activity and influence the bioavailability of iron in the body [101]. Tricin shows chemopreventive effects against breast and colon cancer cells [102].	Citrus flavonoids are available as Citrus Bioflavonoid Caps^®^ and a similar product from soy is available as Phytosoya^®^, SoyChoice^®^
	Oat	Free flavonoids—80% ethanol [103]; Bound flavonoids—Digestion using NaOH, followed by acidification, removal of lipids using hexane and further extraction using ethyl acetate [103].		
Tocols	Barley	Extraction with methanol, followed by drying and extraction using hexane [79]; Saponification using KOH, ethanol, NaCl and pyrogallol at 70 °C for 25 min. This is followed by cooling on ice bath and extraction using hexane:ethyl acetate (9:1) [76,77].	Tocols are generically known as Vitamin E and exhibit antioxidant activity. Tocotrienols demonstrate reduction in serum total cholesterol and LDL cholesterol levels *in vivo* [104].	
	Oat	Extraction techniques used are same as those for barley [76,79,81].		
Proteins	Barley	Alkaline extraction using NaOH and addition of HCl at pH 4.6 for precipitation of protein [105]; A peptide lunasin has been isolated from barley with phosphate-buffered saline supplemented with protease inhibitor cocktail, followed by column and immunoaffinity chromatography [89].	Hydrolyzed peptides from barley proteins have demonstrated anti-oxidant, antihypertensive and anti-diabetic effects *in vitro* [105]. The peptide lunasin is a cancer preventive agent and is bioavailable [89]. Hydrolysates from oat proteins demonstrate ACE-I enzyme inhibitory activity *in vitro* [88].	Milk derived peptides is available as Calpis^®^ and a sardine fish derived peptide product is available as Valtyron^®^, (Source—Fish) Valtyron^®^
	Oat	Alkaline extraction followed by isoelectric precipitation [88].		

## 6. Novel Processing Technologies

There has been a recent surge in the use of alternative food processing techniques such as ultrasound-assisted extraction (UAE), supercritical fluid extraction and accelerated-solvent extraction for extraction of nutraceuticals from plants. The use of novel food processing techniques have some advantages in being more environmental friendly, decreasing solvent consumption, shortening extraction time and giving higher yield than the conventional methods of extraction [106].

UAE is one such potentially useful technology that holds promise for new commercial extraction opportunities and processes in future. It requires no complex instruments and is relatively low cost. In addition to enhancing the extraction efficiency and rate, ultrasonics also holds the potential for achieving simultaneous extraction and encapsulation of extracted compounds and targeted hydroxylation of polyphenolics and carotenoids to enhance bioactivity [107]. Phenolic compounds have been extracted from wheat bran using UAE [108]. The temperature and ethanol concentration required for extraction was 60 °C and 64% respectively, whereas the time required was 25 min. A total phenolic content of 3.12 ± 0.03 mg gallic acid equivalents (GAE)/g bran was obtained using ultrasound extraction which proved it to be as effective as Soxhlet extraction that yielded 2.2–3.2 mg GAE/g bran after treatment for 15 h. In another study, a comparable yield of arabinoxylans and phenolics-rich heteroxylans was achieved using conventional method of extraction and ultrasound assisted extraction [109]. However, UAE not only shortened the time from 60 min required for classical extraction to 5 min, but also lowered the consumption of NaOH used for extraction making it potentially applicable in cosmetics, pharmacy and food industry. Apart from phenolics and polysaccharides, high intensity ultrasound-assisted extraction has been used for extraction of oil from soybeans using the solvents hexane, isopropanol and a 3:2 hexane-isopropanol mixture [110]. Ultrasound in combination with mixed solvent (isopropanol-hexane) resulted in higher yields of oil as compared to non-treated samples. No significant differences in fatty acid composition of the ultrasonicated soybean oil were detected by gas chromatographic analysis. This method was also found to reduce the time required for extraction of edible oil, and hence might help in improving the throughput in commercial oil production processes. UAE has also been demonstrated to be an efficient process in extraction of high molecular β-glucans from a waxy genotype of barley [111]. The results have been attributed to the mechanical effects caused by ultrasonically induced cavitation, which causes an increase in permeability of plant cells by disruption of cell walls, and improves mass transfer limitations of the extraction process. The study inferred that the energy (controlled by time and amplitude) to be used in the UAE process depended on the features of the desired product, in terms of its β-glucan content and molecular weight. Low intensity treatment (250–425 kJ/L) allowed to obtain β-glucan with high MW (>400 kDa) but extraction yields below 50%, whereas high intensity treatment increased the yield of β-glucan while depolymerizing the product, which in some cases might be the purpose.

Pressurized liquid extraction (PLE) also known under the trade name of accelerated solvent extraction (ASE) is a relatively new technology, used for extraction of phytochemicals from natural sources. PLE is a solid-liquid extraction that uses solvents at a high pressure allowing application of temperature to the solvents above their boiling points, PLE was used as a rapid method for extraction of tocopherols and tocotrienols from cereals, followed by their detection using electrospray ionisation-mass spectrometry [112]. Methanol at a temperature of 50 °C, a pressure of 11 MPa and one cycle of extraction with an extraction (static) time of 5 min was found to provide the best results. PLE has also been used for extracting polar lipids and non-polar lipids like phytosterols from ground corn kernels and rolled oats [113]. With both corn and oats, increasing solvent polarity resulted in increasing yield, and for each individual solvent more lipid was extracted at 100 °C than at 40 °C with a static time of 10 min at 7 MPa. In all these studies, the combined use of high pressures (3.3–20.3 MPa) and temperatures (40–200 °C) provides faster extraction process that lowers the solvent consumption along with providing automation and speed. High temperature and pressure improves analyte solubility and desorption kinetics from matrices. Therefore extraction solvents including water that show low efficiency in extracting phytochemicals at low temperatures may be more efficient at elevated PLE temperatures.

Another technique called supercritical fluid extraction (SFE) involves use of fluids in their supercritical (SC) state, which is achieved when the temperature and pressure of a substance is raised over its critical value. This is where the SFE is different from PLE. The supercritical fluid has characteristics of both gases and liquids. A SFE was conducted using carbon dioxide (CO_2_) for extraction of oil from millet bran [114]. In this process, CO_2_ was employed as the supercritical fluid as it has low critical temperature (31.1 °C) and pressure (7.28 MPa). Composition of the crude SC-CO_2_ oil, *i.e.*, fatty acid profile, amount of unsaponifiables and tocopherols was similar to the crude oil obtained by petroleum ether extraction. Oxidative stability of the SC-CO_2_ oil was only slightly lower than that obtained by conventional process. Moreover, the products obtained from SC-CO_2_ are completely free of solvent residues, as opposed to conventionally extracted products. SC-CO_2_ has also been used for extraction of oil and lipids from oat [69] and rice bran [115]. The composition of total fatty acids was found to be similar in SC-CO_2_ oil and the oil extracted by conventional process using hexane. In addition to use of CO_2_ as a supercritical fluid, water as a subcritical fluid is of interest too because of its non-toxic nature and cheap and easy availability. Subcritical water (SCW) or pressurized hot water is being considered as ‘green solvent’ by many authors. It is a new and promising technique for extraction of value added compounds from plant or food matrices [116]. SCW refers to the water in liquid state in the range of 100 °C (boiling point) to 374 °C (critical point) by application of pressure. Density, viscosity, surface tension and diffusion of water change dramatically when changing pressure, and specially temperature. This has an effect on the mass transfer and diffusivity of compounds from the vegetal matrix into the solution. Recently, SCW has been used for extraction of β-glucan from barley [100]. The process dramatically reduced the extraction time and increased the molecular weight by more than three times compared to the conventional processes. Optimal conditions for extraction using SCW were 155 °C, 18 min and 50 bar with an extraction yield of 53.7% and molecular weight of 200 kDa. Both supercritical and subcritical fluid extractions are performed in the absence of both light and air, which significantly reduce the degradation and oxidation processes in comparison to other extraction techniques. However, due to the application of high pressure in these techniques, the requirements for instrumentation are high and the cost of these methods on industrial scale is high.

Microwave assisted extraction (MAE) is a process utilizing microwave energy to facilitate partition of analytes from the sample matrix into the solution. Since water within the plant matrix absorbs microwave energy, cell disruption is promoted by internal superheating which facilitates desorption of chemicals from the matrix thereby improving the recovery of nutraceutical compounds. Though MAE has not been used on cereal matrices, it has been used for extraction of small molecule phenolic compounds such as gallic acid, protocatechuic acid, chlorogenic acid and caffeic acid from a herb species *E. ulmodies* [117]. Isoflavones have been extracted from soy using MAE at 50 °C in 20 min with 50% ethanol being used as the extracting solvent [118]. No degradation of isoflavones was observed in this process and a high reproducibility was achieved. With 75% recovery of total isoflavones, this technique was comparable to ultrasound assisted extraction of isoflavones from soy. Therefore, this technique has the potential to be used as a quick and cheap extraction method for bioactives from cereal sources.

## 7. Conclusions

Oat and barley are cereals that are cultivated all over the world. In addition to being nutritional sources of carbohydrates, proteins, fiber and nutrients like vitamins, sodium and magnesium, they also play a role in prevention of chronic diseases such as diabetes, coronary heart diseases and cancer. The health benefits provided by these cereals are due to the presence of a number of bioactive compounds, present in the different parts of the grain. Some well-known classes of compounds such as dietary fiber (mostly β glucan), phenolics and tocols as well as lipid classes such as phytosterols, proteins and bioactive peptides from oat and barley have been extracted and characterised in various ways in the last two decades. Many of the compounds are bound to the matrix of the grain, which makes their extraction difficult. Use of novel food processing techniques such as ultrasound-assisted, pressurized liquid and enzyme-assisted extraction, might provide more efficient ways for extraction of naturals and novel phytochemicals from oat and barley matrices. This would replace the need for use of conventional procedures that are very time consuming and require huge amounts of energy (heating and stirring). Based on epidemiological studies and biologically plausible mechanisms, scientific evidence has shown that consumption of oat and barley provide health benefits in terms of reduced rates of oxidative stress, chronic age related diseases and various forms of cancer. They may also help regulate blood glucose levels. However, the bioactive capacities of oat and barley and their products, have mostly been determined by *in vitro* assay methods. This implies the need for more *in vivo* studies, and determination of the bioavailability of these cereal based antioxidants in the body. In parallel to this, more information is needed on the complex mechanisms involved in the protective ability of these cereals. This will help in preparing strong and convincing arguments for an increased consumption of oat and barley by the people and to provide better information about their health benefits and to develop new health claims in the future.

Thus, oat and barley are sources of a number of protective bioactive components. Some components in the whole grain of these cereals such as bran or germ may be more important in this protection and should be retained in food processing. Research and development is needed to determine efficient and cost effective ways to fractionate oat and barley, to capture these components in fractions that can be incorporated in food systems. Further work is needed to confirm health benefits of whole grains, develop processing techniques that will improve palatability of wholegrain products and educate consumers about their benefits.

## References

[1] Trowell H. (1972). Ischemic heart disease and dietary fiber. Am. J. Clin. Nutr..

[2] Anderson J.W. (2003). Whole grains protect against atherosclerotic cardiovascular disease. Proc. Nutr. Soc..

[3] Chatenoud L., Tavani A., la Vecchia C., Jacobs D.R., Negri E., Levi F., Franceschi S. (1998). Whole grain food intake and cancer risk. Int. J. Cancer.

[4] Venn B.J., Mann J.I. (2004). Cereal grains, legumes and diabetes. Eur. J. Clin. Nutr..

[5] Slavin J.L. (2005). Dietary fiber and body weight. Nutrition.

[6] Slavin J.L., Martini M.C., Jacobs D.R., Marquart L. (1999). Plausible mechanisms for the protectiveness of whole grains. Am. J. Clin. Nutr..

[7] Slavin J. (2003). Why whole grains are protective: Biological mechanisms. Proc. Nutr. Soc..

[8] EUROSTAT (2014) Crop Products-Annual Data. http://ec.europa.eu/eurostat/data/database.

[9] Baik B.K., Ullrich S.E. (2008). Barley for food: Characteristics, improvement, and renewed interest. J. Cereal Sci..

[10] Biel W., Bobko K., Maciorowski R. (2009). Chemical composition and nutritive value of husked and naked oats grain. J. Cereal Sci..

[11] Webster F.H. (1996). Oats. Cereal Grain Quality.

[12] Yao N., Jannink J.L., Alavi S., White P.J. (2006). Physical and sensory characteristics of extruded products made from two oat lines with different β-glucan concentrations. Cereal Chem..

[13] Flander L., Salmenkallio-Marttila M., Suortti T., Autio K. (2007). Optimization of ingredients and baking process for improved wholemeal oat bread quality. LWT-Food Sci. Technol..

[14] Bhatty R.S., MacGregor A.W., Bhatty R.S. (1993). Nonmalting uses of barley. Barley: Chemistry and Technology.

[15] Newman R.K., Newman C.W. (2008). Barley for Food and Health: Science, Technology, and Products.

[16] Adil G. (2012). Whole-grain cereal bioactive compounds and their health benefits: A review. J. Food Process. Technol..

[17] Garsed K., Scott B.B. (2007). Can oats be taken in a gluten-free diet? A systematic review. Scand. J. Gastroenterol..

[18] Liu S., Stampfer M.J., Hu F.B., Giovannucci E., Rimm E., Manson J.E., Willett W.C. (1999). Whole-grain consumption and risk of coronary heart disease: Results from the Nurses’ Health Study. Am. J. Clin. Nutr..

[19] Munter J.S., Hu F.B., Spiegelman D., Franz M., van Dam R.M. (2007). Whole grain, bran, and germ intake and risk of type 2 diabetes: A prospective cohort study and systematic review. PLoS Med..

[20] Tighe P., Duthie G., Vaughan N., Brittenden J., Simpson W.G., Duthie S., Thies F. (2010). Effect of increased consumption of whole-grain foods on blood pressure and other cardiovascular risk markers in healthy middle-aged persons: A randomized controlled trial. Am. J. Clin. Nutr..

[21] Liu R.H. (2004). Potential synergy of phytochemicals in cancer prevention: Mechanism of action. J. Nutr..

[22] Anderson J.W., Gilinsky N.H., Deakins D.A., Smith S.F., O’Neal D.S., Dillon D.W., Oeltgen P.R. (1991). Lipid responses of hypercholesterolemic men to oat-bran and wheat-bran intake. Am. J. Clin. Nutr..

[23] Åman P. (2006). Cholesterol-lowering effects of barley dietary fiber in humans: Scientific support for a generic health claim. Scand. J. Food Nutr..

[24] Regand A., Chowdhury Z., Tosh S.M., Wolever T.M.S., Wood P. (2011). The molecular weight, solubility and viscosity of oat beta-glucan affect human glycemic response by modifying starch digestibility. Food Chem..

[25] Wood P.J., Siddiqui I.R., Paton D. (1978). Extraction of high-viscosity gums from oats. Cereal Chem..

[26] Bhatty R.S. (1993). Extraction and enrichment (1 leads to 3), (1 leads to 4)-beta-d-glucan from barley and oat brans. Cereal Chem..

[27] Bhatty R.S. (1995). Laboratory and pilot plant extraction and purification of β-glucans from hull-less barley and oat brans. J. Cereal Sci..

[28] Burkus Z., Temelli F. (1998). Effect of extraction conditions on yield, composition, and viscosity stability of barley β-glucan gum. Cereal Chem..

[29] Irakli M., Biliaderis C.G., Izydorczyk M.S., Papadoyannis I.N. (2004). Isolation, structural features and rheological properties of water-extractable β-glucans from different Greek barley cultivars. J. Sci. Food Agric..

[30] Temelli F. (1997). Extraction and functional properties of barley β-glucan as affected by temperature and pH. J. Food Sci..

[31] Dawkins N.L., Nnanna I.A. (1993). Oat gum and β glucan extraction from oat bran and rolled rats: Temperature and pH effects. J. Food Sci..

[32] Benito-Román O., Alonso E., Lucas S. (2011). Optimization of the β-glucan extraction conditions from different waxy barley cultivars. J. Cereal Sci..

[33] Gangopadhyay N., Hossain M.B., Rai D.K., Brunton N.P. (2015). Optimisation of yield and molecular weight of β-glucan from barley flour using response surface methodology. J. Cereal Sci..

[34] Morgan K.R., Ofman D.J. (1998). Glucagel, a gelling β-glucan from barley. Cereal Chem..

[35] Clarke A.E., Stone B.A. (1966). Enzymic hydrolysis of barley and other beta-glucans by a beta-(1→4)-glucan hydrolase. J. Biochem..

[36] Wood P.J., Weisz J., Blackwell B.A. (1991). Molecular characterization of cereal β-d-glucans. Structural analysis of oat β-d-glucan and rapid structural evaluation of β-d-glucans from different sources by high-performance liquid chromatography of oligosaccharides released by lichenase. Cereal Chem..

[37] Edney M.J., Marchylo B.A., MacGregor A.W. (1991). Structure of total barley beta-glucan. J. Inst. Brew..

[38] Wood P.J., Weisz J., Blackwell B.A. (1994). Structural studies of (1→3),(1→4)-beta-d-glucans by ^13^C-nuclear magnetic resonance spectroscopy and by rapid analysis of cellulose-like regions using high-performance anion-exchange chromatography of oligosaccharides released by lichenase. Cereal Chem..

[39] Jiang G., Vasanthan T. (2000). MALDI-MS and HPLC quantification of oligosaccharides of lichenase-hydrolyzed water-soluble β-glucan from ten barley varieties. J. Agric. Food Chem..

[40] Johansson L., Virkki L., Maunu S., Lehto M., Ekholm P., Varo P. (2000). Structural characterization of water soluble β-glucan of oat bran. Carbohydr. Polym..

[41] Johansson L., Tuomainen P., Ylinen M., Ekholm P., Virkki L. (2004). Structural analysis of water-soluble and-insoluble β-glucans of whole-grain oats and barley. Carbohydr. Polym..

[42] Ryu J.H., Lee S., You S., Shim J.H., Yoo S.H. (2012). Effects of barley and oat β-glucan structures on their rheological and thermal characteristics. Carbohydr. Polym..

[43] Visioli F., Galli C. (2001). The role of antioxidants in the Mediterranean diet. Lipids.

[44] Mazid M., Khan T.A., Mohammad F. (2011). Role of secondary metabolites in defense mechanisms of plants. Biol. Med..

[45] Duthie G.G., Duthie S.J., Kyle J.A. (2000). Plant polyphenols in cancer and heart disease: Implications as nutritional antioxidants. Nutr. Res. Rev..

[46] Peterson D.M., Emmons C.L., Hibbs A.H. (2001). Phenolic antioxidants and antioxidant activity in pearling fractions of oat groats. J. Cereal Sci..

[47] Sosulski F., Krygier K., Hogge L. (1982). Free, esterified, and insoluble-bound phenolic acids. 3. Composition of phenolic acids in cereal and potato flours. J. Agric. Food Chem..

[48] Durkee A.B., Thivierge P.A. (1977). Ferulic acid and other phenolics in oat seeds (*Avena sativa* L. var *Hinoat*). J. Food Sci..

[49] Renger A., Steinhart H. (2000). Ferulic acid dehydrodimers as structural elements in cereal dietary fiber. Eur. Food Res. Technol..

[50] Collins F.W., McLachlan D.C., Blackwell B.A. (1991). Oat phenolics: Avenalumic acids, a new group of bound phenolic acids from oat groats and hulls. Cereal Chem..

[51] Bonoli M., Marconi E., Caboni M.F. (2004). Free and bound phenolic compounds in barley (*Hordeum vulgare* L.) flours: Evaluation of the extraction capability of different solvent mixtures and pressurized liquid methods by micellar electrokinetic chromatography and spectrophotometry. J. Chromatogr. A.

[52] Verardo V., Serea C., Segal R., Caboni M.F. (2011). Free and bound minor polar compounds in oats: Different extraction methods and analytical determinations. J. Cereal Sci..

[53] Verardo V., Bonoli M., Marconi E., Caboni M.F. (2008). Distribution of bound hydroxycinnamic acids and their glycosyl esters in barley (*Hordeum vulgare* L.) air-classified flour: Comparative study between reversed phase-high performance chromatography–mass spectrometry (RP-HPLC/MS) and spectrophotometric analysis. J. Agric. Food Chem..

[54] Holtekjølen A.K., Kinitz C., Knutsen S.H. (2006). Flavanol and bound phenolic acid contents in different barley varieties. J. Agric. Food Chem..

[55] Yu J., Vasanthan T., Temelli F. (2001). Analysis of phenolic acids in barley by high-performance liquid chromatography. J. Agric. Food Chem..

[56] Collins F.W. (1989). Oat phenolics: Avenanthramides, novel substituted *N*-cinnamoylanthranilate alkaloids from oat groats and hulls. J. Agric. Food Chem..

[57] Dimberg L.H., Theander O., Lingnert H. (1993). Avenanthramides-a group of phenolic antioxidants in oats. Cereal Chem..

[58] Dimberg L.H., Molteberg E.L., Solheim R., Frølich W. (1996). Variation in oat groats due to variety, storage and heat treatment. I: Phenolic compounds. J. Cereal Sci..

[59] Hitayezu R., Baakdah M.M., Kinnin J., Henderson K., Tsopmo A. (2015). Antioxidant activity, avenanthramide and phenolic acid contents of oat milling fractions. J. Cereal Sci..

[60] Emmons C.L., Peterson D.M. (1999). Antioxidant activity and phenolic contents of oat groats and hulls. Cereal Chem..

[61] Ishihara A., Kojima K., Fujita T., Yamamoto Y., Nakajima H. (2014). New series of avenanthramides in oat seed. Biosci. Biotechnol. Biochem..

[62] Clifford M.N. (2000). Anthocyanins-nature, occurrence and dietary burden. J. Sci. Food Agric..

[63] Rice-Evans C., Miller N., Paganga G. (1997). Antioxidant properties of phenolic compounds. Trends Plant Sci..

[64] Collins F.W., Webster F.H., Webster F.H. (1986). Oat phenolics: Structure, occurrence, and function. Oats: Chemistry and Technology.

[65] McMurrough I. (1981). High-performance liquid chromatography of flavonoids in barley and hops. J. Chromatogr. A.

[66] Klausen K., Mortensen A.G., Laursen B., Haselmann K.F., Jespersen B.M., Fomsgaard I.S. (2010). Phenolic compounds in different barley varieties: Identification by tandem mass spectrometry (QStar) and NMR; quantification by liquid chromatography triple quadrupole-linear ion trap mass spectrometry (Q-Trap). Nat. Prod. Commun..

[67] Zhou M., Robards K., Glennie-Holmes M., Helliwell S. (1999). Oat lipids. J. Am. Oil Chem. Soc..

[68] Price P.B., Parsons J. (1979). Distribution of lipids in embryonic axis, bran-endosperm, and hull fractions of hulless barley and hulless oat grain. J. Agric. Food Chem..

[69] Fors S.M., Eriksson C.E. (1990). Characterization of oils extracted from oats by supercritical carbon dioxide. Lebensm. Wiss. Technol..

[70] Barnes P.J., Holas J., Kratochvil J. (1983). Cereal tocopherols. Progress in Cereal Chemistry and Technology, Proceedings of 7th World Cereal and Bread Congress.

[71] Lampi A.M., Moreau R.A., Piironen V., Hicks K.B. (2004). Pearling barley and rye to produce phytosterol-rich fractions. Lipids.

[72] Moreau R.A., Hicks K.B. (2013). Removal and isolation of germ-rich fractions from hull-less barley using a Fitzpatrick comminuting mill and sieves. Cereal Chem..

[73] Piironen V., Toivo J., Lampi A.M. (2002). Plant sterols in cereals and cereal products. Cereal Chem..

[74] Lagarda M.J., Garcia-Llatas G., Farré R. (2006). Analysis of phytosterols in foods. J. Pharm. Biomed. Anal..

[75] AbuMweis S.S., Marinangeli C.P., Frohlich J., Jones P.J. (2014). Implementing Phytosterols into Medical Practice as a Cholesterol-Lowering Strategy: Overview of Efficacy, Effectiveness, and Safety. Can. J. Cardiol..

[76] Peterson D.M., Jensen C.M., Hoffman D.L., Mannerstedt-Fogelfors B. (2007). Oat tocols: Saponification *vs.* direct extraction and analysis in high-oil genotypes. Cereal Chem..

[77] Panfili G., Fratianni A., Criscio T.D., Marconi E. (2008). Tocol and β-glucan levels in barley varieties and in pearling by-products. Food Chem..

[78] Lásztity R., Berndorfer-Kraszner E., Huszár M. (1980). On the Presence and Distribution of Some Bioactive Agents in Oat Varieties. Cereals for Food and Beverages.

[79] Peterson D.M., Qureshi A.A. (1993). Genotype and environment effects on tocols of barley and oats. Cereal Chem..

[80] Panfili G., Fratianni A., Irano M. (2003). Normal phase high-performance liquid chromatography method for the determination of tocopherols and tocotrienols in cereals. J. Agric. Food Chem..

[81] Peterson D.M. (1995). Oat tocols: Concentration and stability in oat products and distribution within the kernel. Cereal Chem..

[82] Shewry P.R., Napier J.A., Tatham A.S. (1995). Seed storage proteins: Structures and biosynthesis. Plant Cell..

[83] Cunsolo V., Muccilli V., Saletti R., Foti S. (2012). Mass spectrometry in the proteome analysis of mature cereal kernels. Mass Spectrom. Rev..

[84] Wu Y.V., Sexson K.R., Cavins J.F., Inglett G.E. (1972). Oats and their dry-milled fractions. Protein isolation and properties of four varieties. J. Agric. Food Chem..

[85] Howard K.A., Gayler K.R., Eagles H.A., Halloran G.M. (1996). The relationship between D hordein and malting quality in barley. J. Cereal Sci..

[86] Wang C., Tian Z., Chen L., Temelli F., Liu H., Wang Y. (2010). Functionality of barley proteins extracted and fractionated by alkaline and alcohol methods. Cereal Chem..

[87] Cavazos A., Gonzalez de Mejia E. (2013). Identification of bioactive peptides from cereal storage proteins and their potential role in prevention of chronic diseases. Compr. Rev. Food Sci. F..

[88] Cheung I.W., Nakayama S., Hsu M.N., Samaranayaka A.G., Li-Chan E.C. (2009). Angiotensin-I converting enzyme inhibitory activity of hydrolysates from oat (*Avena sativa*) proteins by in silico and *in vitro* analyses. J. Agric. Food Chem..

[89] Jeong H.J., Lam Y., de Lumen B.O. (2002). Barley lunasin suppresses ras-induced colony formation and inhibits core histone acetylation in mammalian cells. J. Agric. Food Chem..

[90] Jeong H.J., Jeong J.B., Hsieh C.C., Hernández-Ledesma B., de Lumen B.O. (2010). Lunasin is prevalent in barley and is bioavailable and bioactive in *in vivo* and *in vitro* studies. Nutr. Cancer.

[91] Benito-Román Ó., Alonso E., Cocero M. (2013). Pressurized hot water extraction of β-glucans from waxy barley. J. Supercrit. Fluids.

[92] Othman R.A., Moghadasian M.H., Jones P.J. (2011). Cholesterol-lowering effects of oat β-glucan. Nutr. Rev..

[93] Nazare J.A., Normand S., Oste Triantafyllou A., Brac de la Perrière A., Desage M., Laville M. (2009). Modulation of the postprandial phase by β-glucan in overweight subjects: Effects on glucose and insulin kinetics. Mol. Nutr. Food Res..

[94] Zhao H., Dong J., Lu J., Chen J., Li Y., Shan L., Gu G. (2006). Effects of extraction solvent mixtures on antioxidant activity evaluation and their extraction capacity and selectivity for free phenolic compounds in barley (*Hordeum vulgare* L.). J. Agric. Food Chem..

[95] Kim H.Y., Kim O.H., Sung M.K. (2003). Effects of phenol-depleted and phenol-rich diets on blood markers of oxidative stress, and urinary excretion of quercetin and kaempferol in healthy volunteers. J. Am. Coll. Nutr..

[96] Vitaglione P., Napolitano A., Fogliano V. (2008). Cereal dietary fiber: A natural functional ingredient to deliver phenolic compounds into the gut. Trends Food Sci. Tech..

[97] Chu Y.F., Wise M.L., Gulvady A.A., Chang T., Kendra D.F., Jan-Willem van Klinken B., O’Shea M. (2013). *In vitro* antioxidant capacity and anti-inflammatory activity of seven common oats. Food Chem..

[98] Bratt K., Sunnerheim K., Bryngelsson S., Fagerlund A., Engman L., Andersson R.E., Dimberg L.H. (2003). Avenanthramides in oats (*Avena sativa* L.) and structure-antioxidant activity relationships. J. Agric. Food Chem..

[99] Chen C.Y., Milbury P.E., Kwak H.K., Collins F.W., Samuel P., Blumberg J.B. (2004). Avenanthramides and phenolic acids from oats are bioavailable and act synergistically with vitamin C to enhance hamster and human LDL resistance to oxidation. J. Nutr..

[100] Žilić S., Hadži-Tašković Šukalović V., Dodig D., Maksimović V., Maksimović M., Basić Z. (2011). Antioxidant activity of small grain cereals caused by phenolics and lipid soluble antioxidants. J. Cereal Sci..

[101] Santos Buelga C., Scalbert A. (2000). Proanthocyanidins and tannin like compounds–nature, occurrence, dietary intake and effects on nutrition and health. J. Sci. Food Agric..

[102] Hudson E.A., Dinh P.A., Kokubun T., Simmonds M.S., Gescher A. (2000). Characterization of potentially chemopreventive phenols in extracts of brown rice that inhibit the growth of human breast and colon cancer cells. Cancer Epidemiol. Biomark. Prev..

[103] Adom K.K., Liu R.H. (2002). Antioxidant activity of grains. J. Agric. Food Chem..

[104] Vasanthi H.R., Parameswari R., Das D.K. (2012). Multifaceted role of tocotrienols in cardioprotection supports their structure: Function relation. Genes Nutr..

[105] Alu'datt M.H., Ereifej K., Abu-Zaiton A., Alrababah M., Almajwal A., Rababah T., Yang W. (2012). Anti-oxidant, anti-diabetic, and anti-hypertensive effects of extracted phenolics and hydrolyzed peptides from barley protein fractions. Int. J. Food Prop..

[106] Wang L., Weller C.L. (2006). Recent advances in extraction of nutraceuticals from plants. Trends Food Sci. Technol..

[107] Vilkhu K., Mawson R., Simons L., Bates D. (2008). Applications and opportunities for ultrasound assisted extraction in the food industry—A review. Innov. Food Sci. Emerg. Technol..

[108] Wang J., Sun B., Cao Y., Tian Y., Li X. (2008). Optimisation of ultrasound-assisted extraction of phenolic compounds from wheat bran. Food Chem..

[109] Hromádková Z., Košt’álová Z., Ebringerová A. (2008). Comparison of conventional and ultrasound-assisted extraction of phenolics-rich heteroxylans from wheat bran. Ultrason. Sonochem..

[110] Li H., Pordesimo L., Weiss J. (2004). High intensity ultrasound-assisted extraction of oil from soybeans. Food Res. Int..

[111] Benito-Román Ó., Alonso E., Cocero M. (2013). Ultrasound-assisted extraction of β-glucans from barley. LWT-Food Sci. Technol..

[112] Bustamante-Rangel M., Delgado-Zamarreno M., Sánchez-Pérez A., Carabias-Martínez R. (2007). Determination of tocopherols and tocotrienols in cereals by pressurized liquid extraction–liquid chromatography–mass spectrometry. Anal. Chim. Acta.

[113] Moreau R.A., Michael J.P., Singh V. (2003). Pressurized liquid extraction of polar and nonpolar lipids in corn and oats with hexane, methylene chloride, isopropanol, and ethanol. J. Am. Oil Chem. Soc..

[114] Devittori C., Gumy D., Kusy A., Colarow L., Bertoli C., Lambelet P. (2000). Supercritical fluid extraction of oil from millet bran. J. Am. Oil Chem. Soc..

[115] Kuk M.S., Dowd M.K. (1998). Supercritical CO_2_ extraction of rice bran. J. Am. Oil Chem. Soc..

[116] Kronholm J., Hartonen K., Riekkola M.L. (2007). Analytical extractions with water at elevated temperatures and pressures. TrAC-Trend Anal. Chem..

[117] Li H., Chen B., Nie L., Yao S. (2004). Solvent effects on focused microwave assisted extraction of polyphenolic acids from Eucommia ulmodies. Phytochem. Anal..

[118] Rostagno M.A., Palma M., Barroso C.G. (2007). Microwave assisted extraction of soy isoflavones. Anal. Chim. Acta.

